# The Future of the Irish Nursing Home Landscape

**DOI:** 10.7759/cureus.80605

**Published:** 2025-03-15

**Authors:** Isabel Ronan, Patrice Crowley, Nicola Cornally, Mohamad M Saab, David Murphy, Sabin Tabirca

**Affiliations:** 1 School of Computer Science and Information Technology, University College Cork, Cork, IRL; 2 Catherine McAuley School of Nursing and Midwifery, University College Cork, Cork, IRL

**Keywords:** ai and machine learning, computational statistics, data analysis, elderly population, hospice and palliative care, ireland, nursing homes, public health informatics, regression analysis, technology development

## Abstract

There is an increasing need to provide care for older adults as Ireland’s population ages. We assess the current Irish nursing home landscape using public datasets and machine learning. We attempt to predict future nursing home needs in Ireland in the year 2050. We also analyse the geographical disparities that exist between different healthcare services in Ireland. Using publicly available data, we analyse nursing home deaths, bed-to-population ratios, and geographic disparities in healthcare accessibility. Furthermore, we use machine learning to forecast population growth in Ireland. We also use an interactive mapping tool to aid healthcare professionals and key stakeholders in understanding the available data and to plan for future resource allocation. We find a strong correlation between the population and the number of nursing home beds. We also find that there are significant geographic disparities between nursing homes, hospices, and hospitals in Ireland. We estimate that 1,252 out of 6,066 (20.64%) nursing home residents received specialist palliative care in 2021. We predict a population increase of approximately 785,695 people (72.6%) by 2050. Our mapping tool was helpful in directing analysis. There is a need for strategic expansion of the Irish nursing home sector and a focus on high-quality general palliative care in nursing homes.

## Introduction

In many Western countries, reliance on family and community to support the older population is diminishing [1]. Increasingly, people are depending on nursing homes to support the care provision for their relatives. Additionally, a significant portion of older people may require specialised care due to health complications, further increasing society’s reliance on these facilities. While the preference of many may still be to live at home independently or with home care, increased needs and reduced levels of independence lead to many older people requiring 24-hour nursing home care [1].

In the past, nursing homes in Ireland have been without coherent policy from the government, leading to an increased demand for private providers of care [2,3]. The failure to plan for the provision of long-term care has led to misalignment in the health system with variations in quality and standards and limited integration of services across sectors. The Health Information and Quality Authority (HIQA) is an independent agency responsible for monitoring social care and healthcare systems in Ireland, while aiming to address variations in care standards [4]. The Health Service Executive (HSE) is the main provider of public health and social care services in Ireland. As a result of these organisations, there is a body of information available about the current state of nursing homes.

In the current study, we aim to analyse present data to predict future nursing home needs in Ireland. Additionally, we directly compare the proximity of nursing homes to hospitals and hospices to highlight the geographical disparities that exist between these services. We attempt to answer these questions using publicly available datasets, distance calculations, and machine learning.

## Materials and methods

The data used in this study was compiled from multiple sources. All data was sourced from May to September 2024. Irish nursing home data, including bed numbers, locations, and costs of care, came from both the website of the Irish HSE and the HIQA Older Person’s Register [5,6]. Geographical JavaScript Object Notation (GeoJSON) for the interactive map came from the OpenStreetMap project, an open-source initiative that uses local knowledge to accurately provide digital maps for use in projects [7]. Population, death, hospital location and hospice location data came from a variety of sources including the Central Statistics Office (CSO), DATA.GOV.IE, the Irish Hospice Foundation, and the Irish Cancer Society [8-11]. We refer to this data as "present data" as it was compiled within the last 10 years. We did not include data from Northern Ireland; we only included data from the Republic of Ireland in our analysis.

Data from both HIQA and the HSE was compared to determine the most up-to-date list of approved nursing homes in Ireland. By matching HIQA IDs, we determined that 530 facilities are approved by both the HSE and HIQA in their most recent reports. We excluded 18 homes from the analysis due to the inability to consolidate their IDs across datasets. The total older population was determined by summing population figures given on a per-county basis. The total number of nursing home beds per county was determined by summing the maximum occupancy of each nursing home within that county. The ratio of people to nursing home beds per county was calculated as r = p/b, where r is the ratio, p is the population, and b is the number of beds. The mean of these ratios is used in subsequent prediction calculations to provide a more equal overview of the country’s future needs; therefore, prediction calculations take the unequal distribution reported by Reddy et al. into account [4].

Distance calculations between data points were made using Irish Transverse Mercator (ITM) coordinates and the Euclidean distance formula. Euclidean distance, which is also known as straight line distance, has been shown to be an adequate proxy for driving travel distance to hospitals [12]. The Euclidean distance formula is outlined below:

$d = \sqrt{(x_2 - x_1)^2 + (y_2 - y_1)^2}$

where (x2, y2) are the ITM coordinates of the nursing home and (x1, y1) are the ITM coordinates of the hospital or hospice. Additionally, we chose to assess nursing home proximity to care based on the 21 km distance reported in mortality research by Nicholl et al. [13].

Using country-wide population results from 1950 to 2024, we used both linear and polynomial regression to predict the Irish population in 2050. We split our data into an 80-20 train-test split using the Scikit-learn and subsequently ran both models to determine which was most suitable for our analysis.

After testing, the chosen model was retrained on all of the data and subsequently used to predict the population in 2050. Using ratios derived from present-day data, we then computed how many nursing home beds would be needed in the future.

In order to increase nurse involvement in the analysis, an easy-to-use interactive map was created to explore the data. The mapping tool was presented to nursing professionals on our research team during an iterative development process. This map contains hospice, hospital, and hospice-friendly locations, along with the locations of all public and private nursing homes in Ireland under the Fair Deal scheme [14]. The nursing homes on this map all contain buttons to locate the closest hospital, the closest hospice, and the closest hospice-friendly location. As outlined by the Irish Hospice Foundation, hospice-friendly locations are places that have an End-of-Life Care Coordinator [10]. There are filters incorporated into the map to show or hide certain groups of locations. Additionally, there is fuzzy search functionality to locate a nursing home by name.

The Leaflet library was used to create the mapping interface along with the filters for each group of data points [15]. The SweetAlert2 library was used to show pop-up messages to the user when they selected nursing home-related action items [16]. The Fuse.js library was used to implement fuzzy searching [17]. All features were created using Vanilla JS.

Python analysis was conducted using Python version 3.12.6 and Pip version 24.2. The Pandas, Matplotlib and Geopandas libraries were used to create graphs for analysis [18-20]. The Scikit-learn library was used to perform machine-learning tasks [21]. Datasets were visualised using a variety of bar, line, and geographical charts. Additionally, both linear and polynomial regression models were used to predict the Irish population in 2050. Further estimates were made using population and nursing home bed ratios derived from 2022 data.

## Results

After all research team members assessed the mapping tool to understand the available data, it was decided to further investigate beds and county populations along with distances from nursing homes to hospitals and hospices using Python. In this section, we present the results of this investigation. All figures below are rounded to two decimal places.

Population and nursing home beds

We base our results on 530 nursing homes in Ireland. In this section, we compare present and future data to both assess the current resources available in Ireland and the country’s future needs.

Nursing home deaths and age distribution (2021)

**Figure 1:**
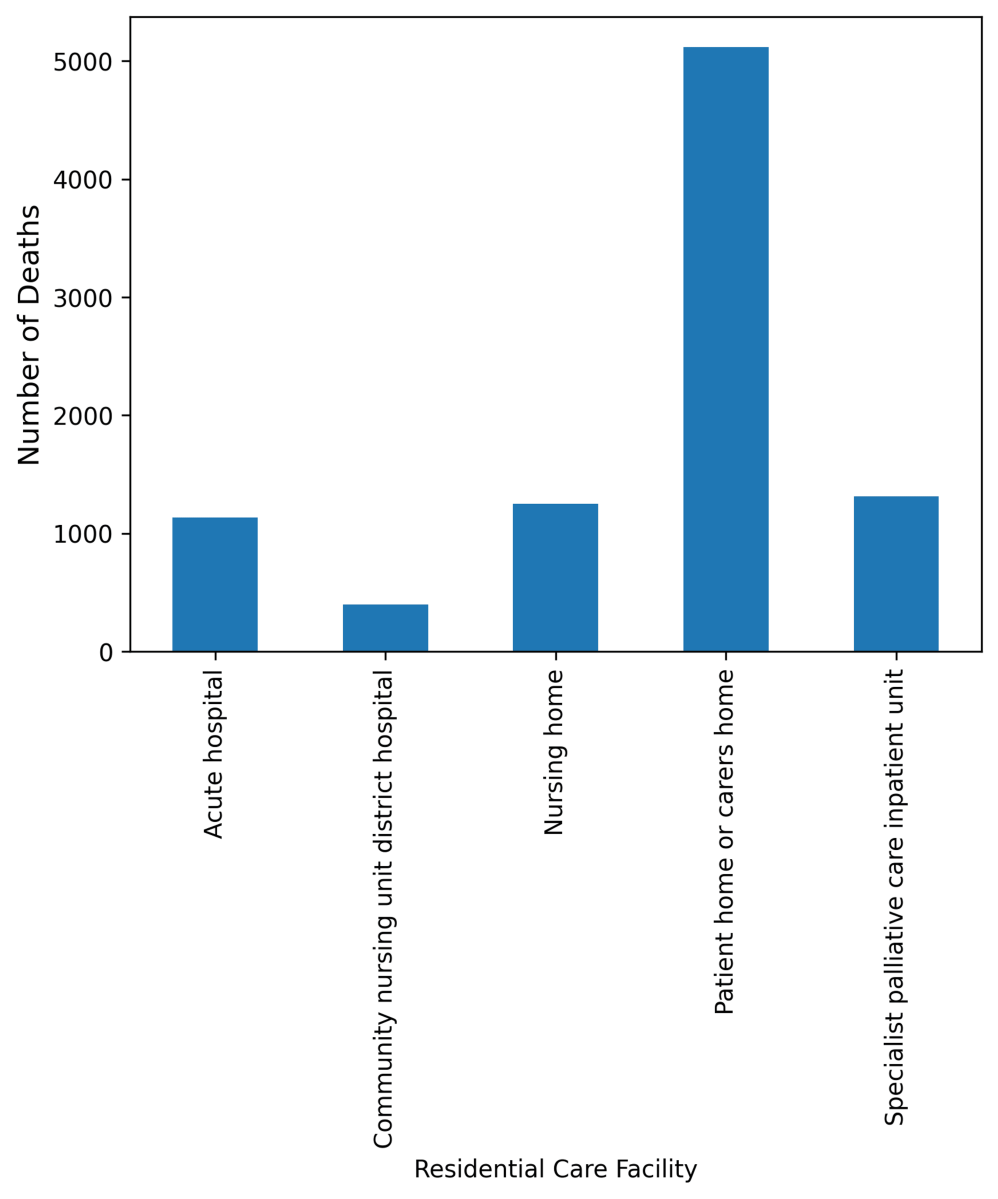
All-Ages Deaths of Those Receiving Specialist Palliative Care in the Community

There were 6,066 nursing home deaths in Ireland in 2021; 5,929 of these deaths were of those aged 65 or over. As shown in Figure 1, the number of deaths for those receiving specialist palliative care in nursing homes is 1,252. From these figures we can posit that 20.64% of nursing home residents received specialist palliative care in 2021.

Correlation between beds and population

**Figure 2:**
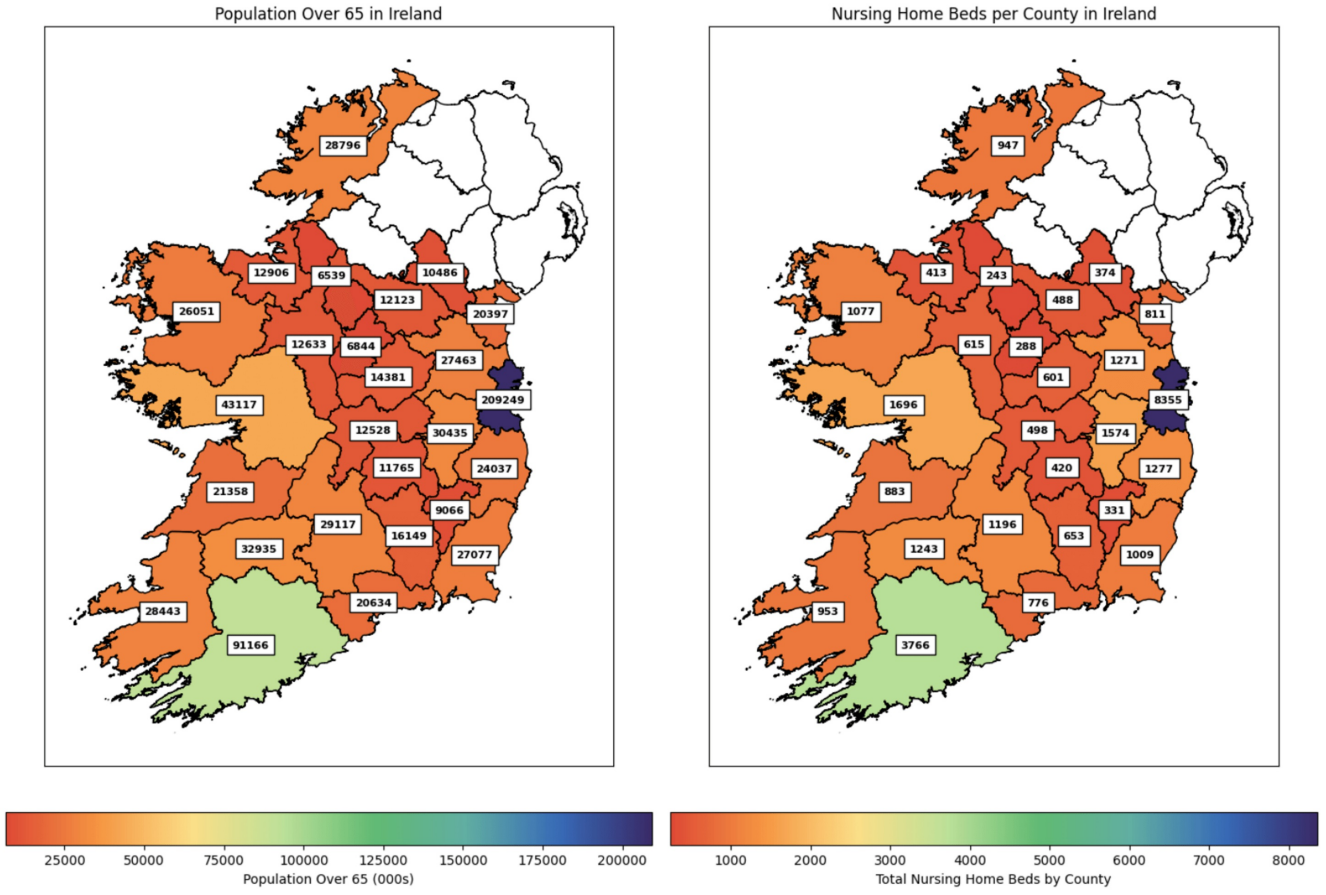
Beds and 65+ Population Per County

The population and nursing home bed numbers per county are represented in Figure 2. We can see from the map that the counties containing Ireland’s most populous cities, Dublin and Cork, contain both a substantial number of people and beds. The Pearson’s correlation coefficient calculated between beds and people is approximately 0.99, indicating a strong positive linear correlation between the two variables. This measure suggests that as the population increases, the number of beds will also need to increase in a proportional way.

The median and mean ratio of nursing home beds to the population are similar, with their values being 25.27 and 25.10 respectively. A small standard deviation of 3.05 reveals a consistent distribution of nursing home beds across the country. An interquartile range of 2.70 reveals uniformity between counties.

Proximity to healthcare facilities

Table 1 outlines the mean and median distances for both hospitals and hospices. We found that 48.11% (255 of 530) of nursing homes are over 21 km from a hospice. Furthermore, 26.6% (141 of 530) of nursing homes are over 21 km from a hospital. These figures reveal the geographic disparity in access to both hospice and hospital care, with nearly half of all nursing homes being a significant distance from hospices. While we can discern, as expected, that this disparity is larger for hospices compared to hospitals, there are still over a quarter of nursing homes that are distant from emergency or specialised care.

**Table 1:** Mean and Median Distances

Calculation	Distance
Nursing Home to Hospice Mean	28.68 km
Nursing Home to Hospice Median	18.40 km
Nursing Home to Hospital Mean	12.76 km
Nursing Home to Hospital Median	7.18 km

Predicted data

**Figure 3:**
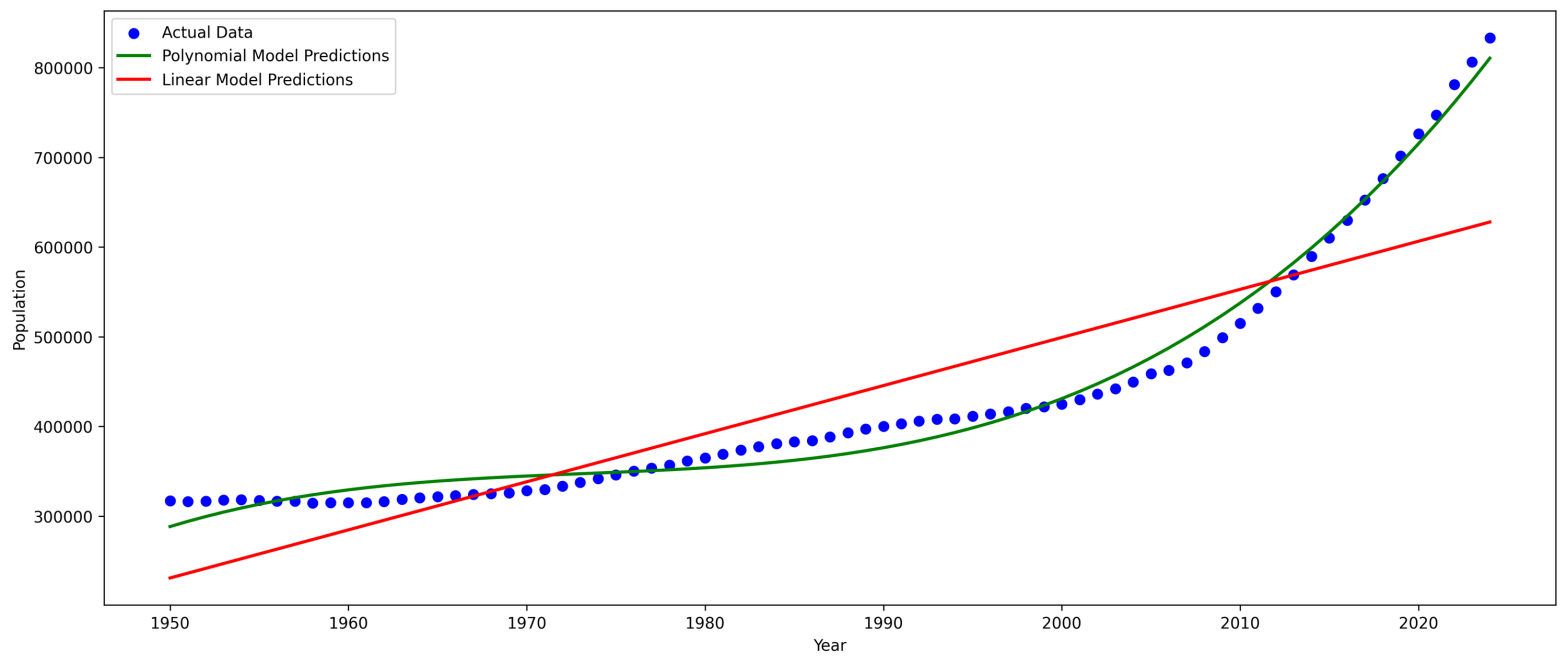
Linear vs Polynomial Regression Models

We used the R-squared measure to assess the performance of our models, with linear and polynomial regression models scoring 0.74 and 0.98 respectively. Therefore, we decided to use the polynomial model in subsequent predictions. Figure 3 shows how the models performed on our data.

The population of older people is projected to increase by 785,695 people by 2050, bringing the total number of individuals aged at least 65 years old to 1,867,956. We found that an estimated 73,915 beds are needed in 2050 to cater to the increasing population, assuming that the current patient-to-bed ratio is sufficient. As there were a total of 31,758 beds reported as of September 2024, an additional 42,157 beds may be required to meet this need.

Mapping tool

Our mapping tool was extremely helpful in comprehending exactly what types of data we had available for our analysis. Insights derived from the map were used to decide the analytical direction of this paper.

## Discussion

As reported by Smith et al., over 75% of beds were provided by private nursing homes in Ireland in 2014 [22]. Their analysis highlights significant inequalities across different healthcare services in Ireland. They also highlight that the government has not based any future health policies on predicted population needs. This imbalance can further challenge the government’s 10-year healthcare reform project, Sláintecare [23]. Ireland’s growing need for nursing home care has also been addressed by Reddy et al. in their work on developing composite indices of geographical access [4]. It was found that there is an inequitable variation in nursing home distribution when detailed needs of the older population are considered. However, Reddy et al. used present data only and did not forecast the future need for nursing home care in a society that is increasingly ageing [4]. Additionally, while Reddy et al.’s work highlighted the accessibility of nursing homes to older people, there was no focus on the proximity of these facilities to other key health services such as acute and specialist palliative care services [4].

As the Irish population ages, more nursing homes are needed to cater to the needs of older people. The number of older people in Ireland has been increasing over the past 75 years. Using historical population data and machine learning, we forecast a significant rise in the number of older people in the country by 2050. As a result of this population increase, we posit that there will be increasing demand for Irish nursing homes.

At present, many nursing homes in Ireland are over 21 km from a hospice or hospital. It has been shown that increased distances between nursing homes and hospitals leads to both increased mortality and a lower likelihood of being brought to the emergency department (ED) for necessary treatment [15,24]. Additionally, the distance from key healthcare services in rural settings can lead to isolation and have a negative impact on the quality of life of nursing home residents [25]. Such undesirable outcomes are not desired by those within such facilities and actually present an interesting contrast between rural and urban nursing home settings.

While we can posit that, for example, rural nursing homes with increased distance from hospitals do not bring their residents as frequently to EDs, avoiding EDs might actually improve a resident’s quality of life. In avoiding EDs, residents may actually circumvent distressing treatment and focus on their own well-being. However, if such a resident feels isolated and does not appreciate the value of rural living, their quality of life can be reduced regardless. Clearly, individual resident preferences are of extreme importance in these situations to improve quality of life, thus making it all the more important to discuss such in the form of advance care plans or advance healthcare directives. 

Potentially, a focus on quality of life and decreasing unnecessary and unwanted hospital admissions may ameliorate geographical issues by improving the well-being of residents. Furthermore, as distances from some rural nursing homes to hospices are large, there may be a need for nursing homes to provide high-quality general palliative care, as opposed to depending on specialist palliative care outreach teams to provide on-site consultation and assessment. However, as outlined above, many of these preferences are individual and will vary from resident to resident. It can still feel isolating for some people to be so far from key services that they may feel in need of.

Our study is not without its limitations. We have not taken Ireland’s health insurance system and income means tests into account in our analysis. The Irish system divides people into categories of subsidised support based on their income [26]. While this categorization can affect access to hospitals, nursing homes and hospices, we believe Ireland’s income-based approach does not hugely influence older peoples’ decisions to go to these facilities, as most of these services have subsidies and schemes in place to ensure somewhat equal access. While we aimed to create as accurate an analysis as possible, we were limited to comparisons between datasets of different years in Ireland (2017-2024). There may be incongruencies between different aspects of the datasets. Although we do not believe any existing disparities between these different datasets are large, we cannot guarantee that our analysis is fully reflective of the current Irish healthcare landscape. Irish nursing homes were also heavily affected by the COVID-19 pandemic; in this paper, we have not addressed the influence of COVID-19 on our analysis. Additionally, our nursing home data came from HSE and HIQA website pages; these website pages list only organization-approved nursing home facilities. As a result, not all Irish nursing homes may be included in these datasets. Furthermore, we assume that the population ratio between each county in Ireland will remain the same between now and 2050, which may not be accurate. We also did not include the analysis of any extra population characteristics that may impact care decisions, such as education, as we did not have such data available. Finally, while we believe our interactive mapping tool allowed for increased data comprehensibility, we have not used any formal usability measures to validate this assumption.

Future work could use standard usability metrics to evaluate our mapping tool. Our analysis could also be improved in future work by sourcing a comprehensive list of nursing homes, whether they are HIQA- and HSE-approved or not. Additionally, our analysis could be repeated in years to come using future population data to compare service and facility development in Ireland.

## Conclusions

In this paper, we outline the analysis of publicly available Irish data regarding nursing homes. An interactive map of our data was created to easily engage with nursing professionals on our research team and to allow increased data accessibility. This map enabled all members of our research team to engage in the generation of insights and areas of exploration. This map subsequently informed further analysis of the same data. We found that the projected increase in Ireland’s population will result in a need for a significant increase in the number of available nursing home beds. Additionally, we found that we need an increase in palliative care trained staff in nursing homes to increase the quality of life of residents and to decrease dependency on healthcare teams which may be too far from rural areas. Future research can support and further develop our findings such that the limitations of our work do not limit the insights gleaned from a constantly evolving Irish healthcare landscape.
